# Supplementary material for: Influence of bending parameters on crystalline undulator radiation peak stability for 530 MeV positron channelling
Source: Eur Phys J D At Mol Opt Phys. 2026 Jul 31;80(8):114. doi: 10.1140/epjd/s10053-026-01227-7 (PMC13427954; doi:10.1140/epjd/s10053-026-01227-7)
Supplement: Supplementary file 1 — (pdf 201 KB) [file 10053_2026_1227_MOESM1_ESM.pdf]

Supplementary Information for: Influence of bending parameters on crystalline undulator radiation peak stability for 530 MeV positron channelling

Matthew D. Dickers[1*], Felipe Fantuzzi[2], Nigel J. Mason[1], Andrei V. Korol[3], Andrey V. Solov'yov[3]

[1]Physics and Astronomy, School of Engineering, Mathematics and Physics, University of Kent, Canterbury, Park Wood Rd, CT2 7NH, United Kingdom.
[2]Chemistry and Forensic Science, School of Natural Sciences, University of Kent, Canterbury, Park Wood Rd, CT2 7NH, United Kingdom.
[3]MBN Research Center, Altenhöferallee 3, 60438 Frankfurt am Main, Germany.

*Corresponding author(s). E-mail(s): M.D.Dickers@kent.ac.uk;
Contributing authors: f.fantuzzi@kent.ac.uk; n.j.mason@kent.ac.uk; korol@mbnexplorer.com; solovyov@mbnresearch.com;

S1 Tables of Physical Parameters

The following tables contain the values associated with the key physical parameters for each amplitude-period combination considered in this study.

Table S1: Bending Parameter C

Bending Amplitude a (Å)	Bending Period λ (µm) 3.5	4.0	4.5	5.0	5.5	6.0	6.5	7.0	7.5	8.0
0.60	0.171	0.131	0.103	0.084	0.069	0.058	0.050	0.043	0.037	0.033
0.87	0.231	0.177	0.139	0.113	0.093	0.078	0.067	0.058	0.050	0.044
1.02	0.290	0.222	0.176	0.142	0.118	0.099	0.084	0.073	0.063	0.056
1.23	0.350	0.268	0.212	0.172	0.142	0.199	0.102	0.088	0.076	0.067
1.44	0.410	0.314	0.248	0.201	0.166	0.139	0.119	0.102	0.089	0.078
1.66	0.473	0.362	0.286	0.232	0.191	0.161	0.137	0.118	0.103	0.090
1.87	0.532	0.408	0.322	0.261	0.216	0.181	0.154	0.133	0.116	0.102
2.08	0.592	0.453	0.358	0.290	0.240	0.201	0.172	0.148	0.129	0.113
2.29	0.652	0.499	0.394	0.319	0.264	0.222	0.189	0.163	0.142	0.125
2.50	0.712	0.545	0.431	0.349	0.288	0.242	0.206	0.178	0.155	0.136

Table S2: Undulator Parameter K_{u}^2

Bending Amplitude a (Å)	Bending Period λ (µm) 3.5	4.0	4.5	5.0	5.5	6.0	6.5	7.0	7.5	8.0
0.60	0.112	0.098	0.087	0.078	0.071	0.065	0.060	0.056	0.052	0.049
0.87	0.151	0.132	0.117	0.106	0.096	0.088	0.081	0.075	0.070	0.066
1.02	0.190	0.166	0.148	0.133	0.121	0.111	0.102	0.095	0.089	0.083
1.23	0.229	0.200	0.178	0.160	0.146	0.134	0.123	0.115	0.107	0.100
1.44	0.268	0.235	0.209	0.188	0.171	0.156	0.144	0.134	0.125	0.117
1.66	0.309	0.270	0.240	0.216	0.197	0.180	0.166	0.155	0.144	0.135
1.87	0.348	0.305	0.271	0.244	0.222	0.203	0.187	0.174	0.162	0.152
2.08	0.387	0.339	0.301	0.271	0.246	0.226	0.209	0.194	0.181	0.169
2.29	0.426	0.373	0.332	0.298	0.271	0.249	0.230	0.213	0.199	0.187
2.50	0.465	0.407	0.362	0.326	0.296	0.272	0.251	0.233	0.217	0.204

Table S3: Combined Undulator Parameter $K^2 = K_{\mathrm{u}}^2 + K_{\mathrm{ch}}^2(1 - C)$

Bending Amplitude a (Å)	Bending Period λ (µm) 3.5	4.0	4.5	5.0	5.5	6.0	6.5	7.0	7.5	8.0
0.60	0.035	0.033	0.032	0.031	0.030	0.030	0.029	0.029	0.029	0.029
0.87	0.044	0.040	0.037	0.035	0.034	0.033	0.032	0.031	0.031	0.030
1.02	0.055	0.049	0.044	0.041	0.038	0.037	0.035	0.034	0.033	0.032
1.23	0.070	0.060	0.053	0.048	0.044	0.042	0.040	0.038	0.036	0.035
1.44	0.088	0.074	0.064	0.057	0.052	0.048	0.045	0.042	0.040	0.039
1.66	0.110	0.090	0.077	0.068	0.061	0.055	0.051	0.048	0.045	0.043
1.87	0.134	0.109	0.092	0.079	0.070	0.063	0.058	0.054	0.050	0.048
2.08	0.161	0.130	0.108	0.093	0.081	0.073	0.066	0.061	0.056	0.053
2.29	0.191	0.153	0.126	0.108	0.094	0.083	0.075	0.068	0.063	0.058
2.50	0.224	0.178	0.146	0.124	0.107	0.094	0.084	0.076	0.070	0.065

Table S4: First Harmonic Position $\hbar\omega_1$

Bending Amplitude a (Å)	Bending Period λ (µm) 3.5	4.0	4.5	5.0	5.5	6.0	6.5	7.0	7.5	8.0
0.60	0.749	0.656	0.584	0.526	0.478	0.438	0.405	0.376	0.351	0.329
0.87	0.746	0.654	0.582	0.524	0.477	0.438	0.404	0.375	0.350	0.329
1.02	0.742	0.651	0.580	0.523	0.476	0.437	0.403	0.375	0.350	0.328
1.23	0.737	0.648	0.578	0.521	0.475	0.436	0.403	0.374	0.349	0.328
1.44	0.730	0.643	0.575	0.519	0.473	0.434	0.402	0.373	0.349	0.327
1.66	0.723	0.638	0.571	0.516	0.471	0.433	0.400	0.372	0.348	0.327
1.87	0.715	0.633	0.567	0.513	0.469	0.421	0.399	0.371	0.347	0.326
2.08	0.706	0.627	0.563	0.510	0.466	0.429	0.397	0.370	0.346	0.325
2.29	0.696	0.620	0.558	0.506	0.464	0.427	0.396	0.369	0.345	0.324
2.50	0.686	0.631	0.553	0.503	0.461	0.425	0.394	0.367	0.344	0.323

Table S5: Acceptance $\mathcal{A}$

Bending Amplitude a (Å)	Bending Period λ (µm) 3.5	4.0	4.5	5.0	5.5	6.0	6.5	7.0	7.5	8.0
0.60	0.858	0.884	0.904	0.920	0.924	0.934	0.935	0.938	0.933	0.938
0.87	0.808	0.855	0.883	0.910	0.921	0.930	0.915	0.928	0.933	0.936
1.02	0.790	0.833	0.880	0.889	0.884	0.904	0.920	0.914	0.920	0.927
1.23	0.727	0.789	0.849	0.870	0.888	0.899	0.905	0.910	0.912	0.921
1.44	0.678	0.763	0.807	0.845	0.868	0.880	0.893	0.910	0.916	0.915
1.66	0.621	0.739	0.787	0.819	0.854	0.872	0.884	0.894	0.907	0.904
1.87	0.581	0.692	0.763	0.791	0.826	0.850	0.875	0.893	0.882	0.892
2.08	0.536	0.659	0.748	0.776	0.810	0.848	0.851	0.880	0.888	0.890
2.29	0.509	0.605	0.699	0.754	0.804	0.827	0.850	0.867	0.873	0.883
2.50	0.440	0.599	0.697	0.737	0.779	0.800	0.859	0.852	0.865	0.879
